# Changes of Brain Structure in Patients With Metastatic Non-Small Cell Lung Cancer After Long-Term Target Therapy With EGFR-TKI

**DOI:** 10.3389/fonc.2020.573512

**Published:** 2021-01-06

**Authors:** Beisheng Yang, Chunli Luo, Min Yu, Lin Zhou, Bo Tao, Biqiu Tang, Ying Zhou, Jiang Zhu, Meijuan Huang, Feng Peng, Yongmei Liu, Yong Xu, Yan Zhang, Xiaojuan Zhou, Jianxin Xue, Yanying Li, Yongsheng Wang, Zhiping Li, You Lu, Su Lui, Youling Gong

**Affiliations:** ^1^ Huaxi MR Research Center (HMRRC), Department of Radiology, West China Hospital of Sichuan University, Chengdu, China; ^2^ Department of Thoracic Oncology and State Key Laboratory of Biotherapy, Cancer Center, West China Hospital, Sichuan University, Chengdu, China; ^3^ Department of Radiation Oncology, Cancer Center, West China Hospital, Sichuan University, Chengdu, China; ^4^ Department of Radiation Oncology, West China Hospital, Sichuan University, Chengdu, China

**Keywords:** non-small cell lung cancer, epidermal growth factor receptor-tyrosine kinase inhibitor, white matter lesion, gray matter, MRI

## Abstract

**Purpose:**

Epidermal growth factor receptor-tyrosine kinase inhibitor (EGFR-TKI) therapy is the routine treatment for patients with metastatic non-small cell lung cancer (NSCLC) harboring positive EGFR mutations. Patients who undergo such treatment have reported cognitive decline during follow-up. This study, therefore, aimed to evaluate brain structural changes in patients receiving EGFR-TKI to increase understanding of this potential symptom.

**Method:**

The medical records of 75 patients with metastatic NSCLC (without brain metastasis or other co-morbidities) who received EGFR-TKI therapy from 2010 to 2017 were reviewed. The modified Scheltens Visual Scale and voxel-based morphometry were used to evaluate changes in white matter lesions (WML) and gray matter volume (GMV), respectively.

**Results:**

The WML scores were higher at the 12-month [8.65 ± 3.86; 95% confidence interval (CI), 1.60–2.35; p < 0.001] and 24-month follow-ups (10.11 ± 3.85; 95% CI, 2.98–3.87; p < 0.001) compared to baseline (6.68 ± 3.64). At the 24-month follow-up, the visual scores were also significantly higher in younger patients (3.89 ± 2.04) than in older patients (3.00 ± 1.78; p = 0.047) and higher in female patients (3.80 ± 2.04) than in male patients (2.73 ± 1.56; p = 0.023). Additionally, significant GMV loss was observed in sub-regions of the right occipital lobe (76.71 voxels; 95% CI, 40.740–112.69 voxels), left occipital lobe (93.48 voxels; 95% CI, 37.48–149.47 voxels), and left basal ganglia (37.57 voxels; 95% CI, 21.58–53.57 voxels) (all p < 0.005; cluster-level false discovery rate < 0.05).

**Conclusions:**

An increase in WMLs and loss of GMV were observed in patients with metastatic NSCLC undergoing long-term EGFR-TKI treatment. This might reflect an unknown side-effect of EGFR-TKI treatment. Further prospective studies are necessary to confirm our findings.

## Introduction

Non-small cell lung cancer (NSCLC) is the leading cause of cancer death worldwide. About 40–50% of Asian patients with NSCLC harbor epidermal growth factor receptor (EGFR) mutations, and distant metastases are observed in nearly 40% of these patients at initial diagnosis (1). The approval of gefitinib, the first-generation EGFR tyrosine kinase inhibitor (TKI), led to the development of molecular targeted therapy for lung cancer (2). Prospective phase III trials have established that EGFR-TKIs are superior to chemotherapy for patients harboring an EGFR mutation (3–6). Therefore, EGFR-TKIs have been recommended as first-line treatment for such patients in clinical guidelines (7, 8). Consequently, EGFR-TKI therapy has been routinely prescribed for patients with EGFR mutations worldwide.

The known side effects of targeted therapy with EGFR-TKIs include rashes, diarrhea, hepatic impairment, mucositis, and interstitial pneumonia (9). However, during routine follow-ups, patients with NSCLC undergoing EGFR-TKI treatment in our department, the Oncology Department of West-China Hospital, have reported cognitive decline after starting EGFR-TKI treatment, a side effect that has not previously been reported. Meanwhile, a recent study evaluated the neuropsychological performance of patients with NSCLC who underwent targeted therapy and reported that depression and/or anxiety were correlated with the treatment, but details regarding drug utilization in this study were not clear (10). Potential reasons for these neuropsychiatric symptoms remain unknown.

Many studies have reported that epidermal growth factor (EGF) is involved in the main biological pathway of neurodevelopment and repair of nerve injury by promoting the proliferation, regeneration, and development of neurons (11–13). Thus, the inhibition of the EGF pathway caused by EGFR-TKIs could negatively influence the differentiation, maturation, and rehabilitation of neural cells, which may lead to chronic cognitive function impairments. In this context, exploring structural changes in patients’ brains before and after EGFR-TKI treatment could help in determining the causes of cognitive decline.

It has been reported that white matter lesions (WMLs) are an indication of cognitive impairment, especially in the elderly and patients with specific comorbidities (14, 15). The loss of gray matter volume (GMV) has also been associated with mild cognitive impairment, including memory loss and attention and language dysfunction (16, 17). MRI-based studies have confirmed that WMLs and gray matter atrophy (GMA) could be the primary imaging correlates of early dementia and mild cognitive impairment in several chronic diseases, including Parkinson’s disease, diabetes mellitus, and Alzheimer’s disease, among others (18–20).

Patients with metastatic NSCLC who undergo EGFR-TKI treatment at our hospital receive routine MRIs. This allowed us to investigate the potential impact of long-term EGFR-TKI therapy on patients’ brains, which has not yet been evaluated. Thus, for the first time, we collected brain MRI images of patients with NSCLC to investigate changes in WMLs and GMV.

## Materials and Methods

This retrospective study was approved by the ethics committee at West China Hospital, Sichuan University and was in full accordance with the International Conference of Harmonization Good Clinical Practice Guidelines. Informed consent was obtained during follow-up, and for those who were lost to follow-up (e.g., death, emigration), we were granted permission by the ethics committee at West China Hospital, Sichuan University for an informed consent waiver.

Patients with pathologically-confirmed metastatic NSCLC between 2010 and 2017 in West China Hospital, Sichuan University were included. The inclusion criteria were as follows: a) positive for EGFR mutation (19 exon deletion or 21 exon L858R mutation detected by amplification refractory mutation system, differential display-polymerase chain reaction, or next generation sequencing), b) received first-generation EGFR-TKIs (gefitinib, erlotinib, or icotinib, according to the Chinese Food and Drug Administration’s approval); and c) brain MRI data available during follow-up at our hospital. Exclusion criteria were as follows: a) suffering from a neurological or psychiatric disease; b) presence of comorbidities that might influence the patient’s brain structure (e.g., type 2 diabetes, hypertension, chronic kidney disease); c) identification of brain metastases during MRI at baseline or anytime during the evaluation period; d) substantial abuse including alcohol or narcotics; and e) any other concurrent systematic therapy (e.g., chemotherapy, anti-angiogenetic therapy, or immunotherapy).

### Treatment and Follow-Up

After EGFR-TKI treatment initiation, objective assessments of all the eligible patients were recorded every 3 months according to the RESIST criteria (21). The patients underwent a brain MRI every 6 months when no neural symptoms or physical signs were observed; otherwise, a brain MRI was performed immediately to rule-out brain metastasis. The EGFR-TKI dose was modified according to the instructions specific to each drug. The duration of the follow-up period was ≥ 24 months.

### Image Acquisition

Image data were retrospectively collected from our hospital’s PACS (Picture Archiving and Communication System). Since there are multiple MRI scanners at our hospital, T2-FLAIR (fluid attenuated inversion recovery) images for WML assessments were acquired using different scanners from two different manufacturers (GE and Siemens) and with varying magnetic field intensities (1.5-T, n = 154 person-time; and 3.0-T, n = 82 person-time). Since the WML diagnostic features were high signal spots in the T2-FLAIR sequence, the evaluation of visual scores were not affected by the different scanners. A high resolution T1WI (1.0 mm/slice) MRI was not routinely used for all the patients due to its extra charge; however, we screened the image data for available high resolution T1WI MRIs before and after about 1-year treatment, which were obtained by the same 3.0 T MRI system. The images of 21 patients (13 by Siemens scanners; 8 by GE scanners) were determined to be suitable for GMV analysis.

### Definitions and Acquisition of White Matter Lesion and Gray Matter Volume

WMLs are regions of white matter that have an abnormal white matter fiber tract, which present as hyperintense regions on MRI T2-FLAIR sequence images with different shapes categorized as: periventricular caps, rims, or halos; subcortical multiple punctuates or patchy lesions; and partially or completely confluent lesions. They are often divided into two broad categories, namely, periventricular WMLs (attached to the ventricular system) and deep WMLs (located at the subcortical white matter area) (22, 23).

WMLs from axial T2-FLAIR images were evaluated using the modified Scheltens Visual Scale (SVS; **Supplementary Material, Section A**), with which periventricular and white matter hyperintensities are semi-quantitively rated. The modified SVS is used to rate WMLs in the periventricular region on a 7-point scale (0–6) and those in the subcortical region on a 25-point scale (0–24) according to the size and number of lesions (24, 25). The modified SVS was used to evaluate the WMLs seen on T2-FLAIR images at baseline and after 12 months and 24 months of EGFR-TKI therapy (one representative patient is shown in **Supplementary Material, Section B**).

Gray matter is a major component of brain parenchyma and consists of neuronal cell bodies, neuropils (dendrites and axons), glial cells, and capillaries. It is distinguished from white matter in that it contains numerous neuronal cell bodies and relatively few myelinated axons. GMV is determined using optimized voxel-based morphometry (VBM) (26), a computational neuroanatomy method that measures the number of voxels of gray matter after separating them from white matter using T1WI.

In this study, the GMV analysis was performed using the Statistical Parametric Mapping Package (SPM8) (http://www.fil.ion.ucl.ac.uk/spm/), with the VBM-based diffeomorphic anatomical registration using the exponentiated lie algebra (VBM-DARTEL) toolbox (27). First, the high-resolution images of all the patients before and after treatment were segmented into gray matter, white matter, and cerebrospinal fluid. Second, the segmented gray matter was smoothed to create the primary DARTAL template. After 18 iterative operations with raw segmented gray matter images, six templates were created and the sixth template, which is considered to have maximum accuracy and sensitivity (28), was registered to the Montreal Neurological Institute space. All the patients’ GMVs before and after treatment were obtained for further statistical analysis.

### Statistical Analysis

The normality of the WML SVS scores was tested using the Shapiro-Wilk test. Paired sample t-tests were used to test differences in the WML scores before and after treatment. Independent sample t-tests were used to evaluate variations in WMLs from baseline according to sex, age, type of mutation, and type of TKI therapy.

Changes in GMV before and after EGFR-TKI therapy were tested using paired sample t-tests (uncorrected p value < 0.001), and corrected using a false discovery rate (FDR) of p < 0.05 at cluster level and peak level. A cluster level test takes into account the size of the cluster that consists of adjacent voxels as test objects, and a cluster size above the voxel’s threshold has a statistical significance suitable for small samples. For the peak level test, each voxel is regarded as an independent test subject, meaning a much stricter FDR is required for viability.

## Results

### Patient Characteristics

The median age of all 75 patents with NSCLC was 60 years (range, 38–71 years) and the majority were women (49/75, 65.3%). Forty-one (54.7%) and 34 (45.3%) patients were positive for EGFR 19 exon deletion and 21 exon L858R transformation, respectively. The median duration of intracranial progression-free survival was 32.0 months (range, 23.0–89.0 months). For the 21 patients included in the GMV analysis, the median age was 59 years (range, 43–70 years) and the majority were female (12/21, 57.1%) (**Table 1**).

**Table 1:** Baseline characteristics of patients in present study.

Baseline characteristics	Patients for WML analysis, number	Patients for GMV analysis, number
Age (years)	60 (range of 38–71)	59 (range of 43–70)
Sex (%)		
Male	26 (34.7)	9 (42.9)
Female	49 (65.3)	12 (57.1)
ECOG performance status (%)		
0	38 (50.7)	10 (47.6)
1	37 (49.3)	11 (52.4)
EGFR mutation (%)		
19 del	41 (54.7)	12 (57.1)
21L858R	34 (45.3)	9 (42.9)
EGFR-TKI (%)		
Gefitinib	30 (40)	13 (61.9)
non-Gefitinib	45 (60)	8 (38.1)
Progression-free survival (months)	32 (range of 23-89)	

Data are median (IQR) or number (%); ECOG, Eastern Cooperative Oncology Group; EGFR, epidermal growth factor receptor; TKI, tyrosine kinase inhibitor.

### Changes in White Matter Lesions

**Figure 1:**
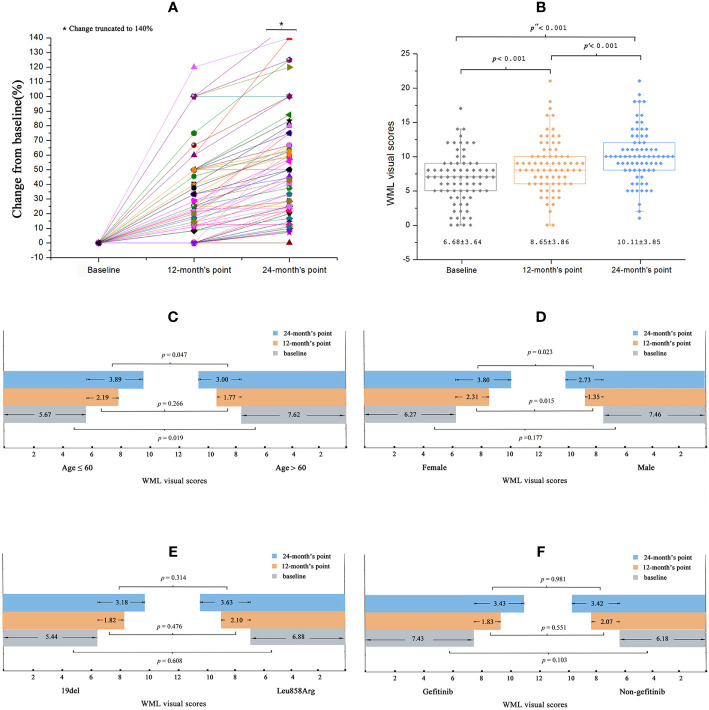
Baseline and changes of WML visual scores in all patients. **(A)** During the treatment of EGFR-TKI, the patient’s WML visual scores increased progressively. **(B)** Comparing to the baseline scores, the scores were significantly changed at the 12-months’ point and changed more obviously at the 24-months’ point. **(C)** Sub-group analysis: the baseline WML visual score was significantly higher in elder patients. The WML visual scores increased more significantly at the 24-month point in younger patients than elder patients. **(D)** There was no difference between the baseline WML visual scores among female and male patients, while the visual scores increased more significantly in female patients at the 12-month’s point and 24-month’s point than that in the male patients. **(E**, **F)** No significant differences of the WML visual scores was observed between EGFR mutation types or EGFR-TKIs the patients receiving.

The SVS scores of the WMLs at baseline varied between 0 and 17.00, and increased at the 12-month and 24-month follow-ups (**Figure 1A**). Compared to baseline (6.68 ± 3.64), the scores were significantly higher at the 12-month [8.65 ± 3.86; 95% confidence interval (CI) 1.56–2.35, p < 0.001] and 24-month (10.11 ± 3.85; 95% CI 2.98–3.87, p < 0.001) follow-ups (**Figure 1B**).

Sub-group analyses showed that the SVS scores at baseline were significantly higher in older patients (> 60 years) than in younger patients (≤ 60 years) (7.62 ± 3.56 vs. 5.67 ± 3.49, respectively; p = 0.019). Compared to older patients, the younger patients also showed significantly higher SVS scores at the 24-month follow-up (3.00 ± 1.78 vs. 3.89 ± 2.04, respectively; p = 0.047) but not at the 12-month (2.19 ± 1.69 vs. 1.77 ± 1.56, respectively; p = 0.266) follow-up (**Figure 1C**). For the SVS scores at baseline, no significant differences were found between female and male patients (6.27 ± 3.37 vs. 7.46 ± 4.05, respectively; p = 0.177). However, SVS scores were significantly higher for female patients than male patients at the 12-month (2.31 ± 1.66 vs. 1.35 ± 1.44, respectively; p = 0.015) and 24-month (3.80 ± 2.04 vs. 2.73 ± 1.56, respectively; p = 0.023) follow-ups (**Figure 1D**). No significant differences in SVS scores were observed according to the different EGFR mutations or EGFR-TKI treatments at baseline or at the 12-month or 24-month follow-ups (all p > 0.05; **Figures 1E, F** **)**.

### Changes in Gray Matter Volume

**Figure 2:**
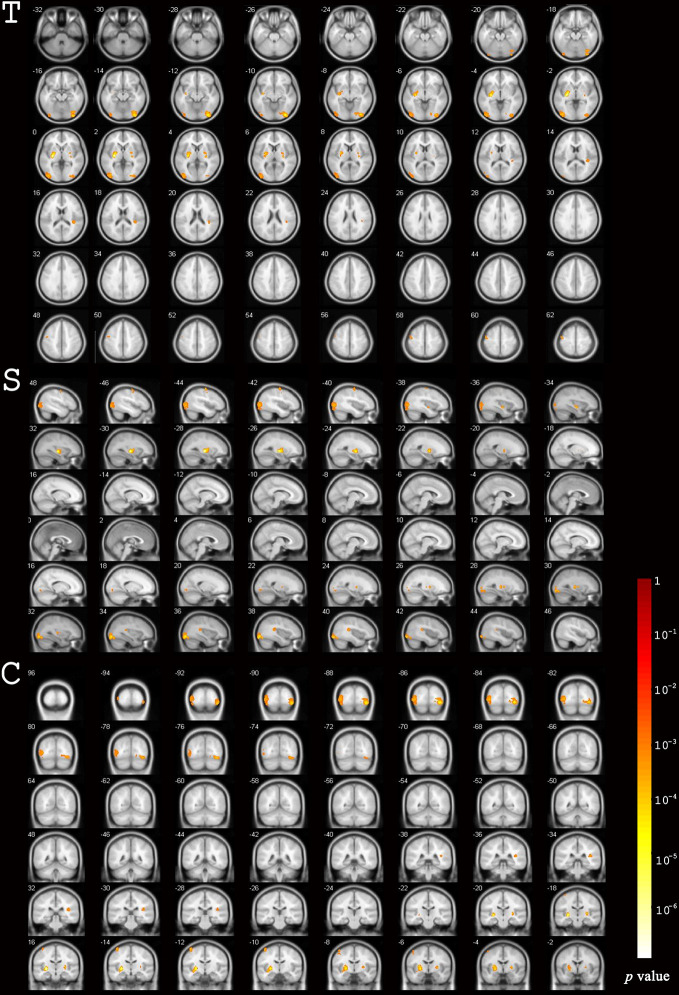
Differences in gray matter volume before and after EGFR-TKI treatment. Significant differences were identified using voxel-based paired sample T-test.

The total GMV of patients was 673.8 ± 58.5 cm^3^ and 667.6 ± 60.3 cm^3^ at baseline and the 12-month follow-up, respectively. Uncorrected GMV loss (p < 0.001) was identified in brain MRIs (**Figure 2**). The total voxel values were clearly lower after EGFR-TKI treatment than at baseline in three main clusters: the sub-regions of the middle and inferior occipital cortex (1,697 voxels); the right middle and inferior occipital cortex extending to the lingual gyrus and entorhinal cortex (1,660 voxels); and the left lentiform nucleus, which included the putamen and pallidum (1,145 voxels) (**Table 2**). Mild GMV loss was observed in the left precentral gyrus, which included part of Brodmann area 6 (321 voxels); two independent clusters at the right lentiform nucleus (142 and 125 voxels), both of which included parts of the putamen and pallidum; and the right insula extending to the superior temporal gyrus (269 voxels).

**Table 2:** Sub-regions with GMV atrophy of patients treated with EGFR-TKIs.

Cluster	Total voxels	Main brain sub-regions					
		Region1	Voxels	Region2	Voxels	Region3	Voxels
1	1697	Occipital_Mid_L	1153	Occipital_Inf_L	377	–	–
2	1660	Occipital_Inf_R	705	Occipital_Mid_R	573	Fusiform_R	247
3	1145	Lentiform Nucleus_L	782	Putamen_L	604	Pallidum_L	174
4	321	Precentral Gyrus_L	211	Brodmann area 6	96	–	–
5	269	Insula_R	109	Temporal_Sup_R	84	–	–
6	142	Lentiform Nucleus_R	115	Putamen_R	106	Pallidum_R	9
7	125	Lentiform Nucleus_R	123	Putamen_R	79	Pallidum_R	45

GMV, gray mater volume.

**Figure 3:**
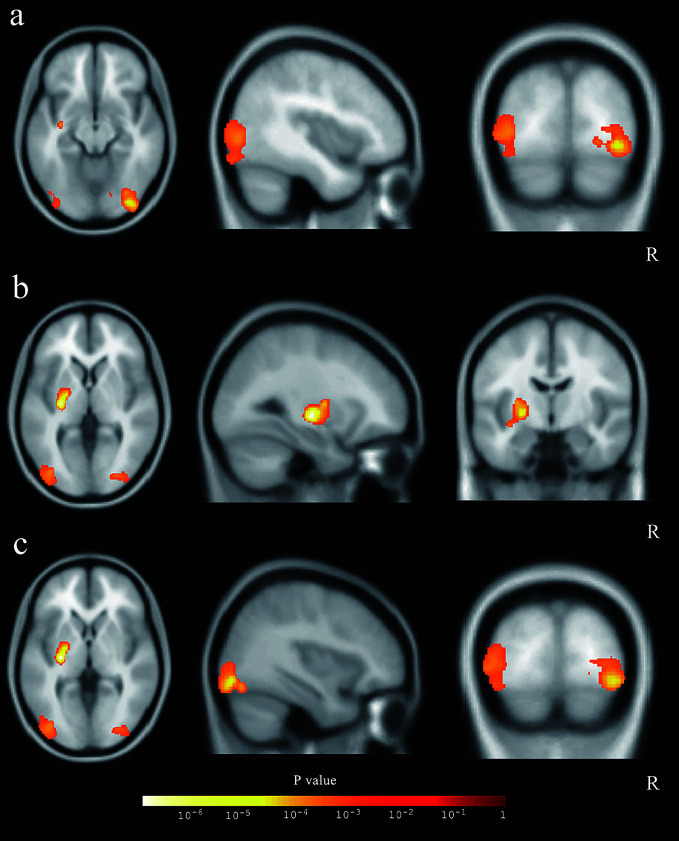
The three main clusters with reduced gray matter volume. The three clusters were showed in axial, sagittal, and coronal positions: **(A)** the right middle and inferior occipital cortex, **(B)** the left lentiform nucleus, and **(C)** the middle and inferior occipital cortex.

After the cluster-level FDR was corrected to p < 0.05, significant GMV loss remained in three main clusters: the right middle and inferior occipital cortex extending to the lingual and entorhinal gyrus, the left lentiform nucleus, and the middle and inferior occipital cortex (p = 0.012, p = 0.003, and p = 0.003, respectively) (**Table 3**, **Figure 3**). The peak-level FDR-corrected analysis showed no significant difference between GMV atrophies at baseline and the 12-month follow-up in these three main clusters (p = 0.054, p = 0.653, and p = 0.885, respectively). The other four clusters were not significant after cluster-level or peak-level FDR correction (all p > 0.05).

**Table 3:** Statistic information of sub-regions with GMV reduction in patients treated with EGFR-TKIs.

Cluster	Cluster-level			Peak-level			X (mm)	Y (mm)	Z (mm)
	Equivk	*p* (un-corr)	*p* (FDR-corr)	T	*p* (uncorr)	*p* (FDR-corr)			
1	1145	0.001	0.012	6.88	0.000	0.054	−29	−17	−2
2	1660	0.000	0.003	4.84	0.000	0.653	35	−86	−11
3	1697	0.000	0.003	3.78	0.001	0.885	−39	−83	0
4	321	0.06	0.387	3.83	0.001	0.885	−44	−12	65
5	142	0.194	0.825	4.09	0.000	0.885	29	−18	5
6	125	0.222	0.825	3.69	0.001	0.885	26	−5	2
7	269	0.082	0.424	3.50	0.001	0.885	41	−33	17

GMV, gray matter volume; EGFR, epidermal growth factor receptor; TKI, tyrosine kinase inhibitor; Equivk, equivalent voxels k; Corr, corrected; FDR, false discovery rate; X, Y, Z, x,y and z axis. respectively.

## Discussion

Previous studies have revealed that the most common adverse effects of EGFR-TKI therapy are skin rashes (31.4%), diarrhea (14.2%), pruritus (6.7%), and hepatic toxicity (3.8%) (9). For the first time, using a series of brain MRIs, significant worsening of WMLs and GMA were observed among patients with advanced-stage NSCLC receiving long-term EGFR-TKI treatment.

The existing literature suggests that EGF is expressed in the cortical plate during neural development, promoting the neurite outgrowth of cortical neurons (11), and the EGFR pathway is linked to multiple nerve cell events, such as proliferation, differentiation, and apoptosis (12). Recently, an EGFR pathway-regulating compound (yhhu-3792) was reported to induce cognitive impairment in mice by inhibiting neural pathways in the hippocampus (29). This microvascular anomaly is believed to be one the principle causes of WMLs, as EGFR and vascular EGF pathways are closely related and share common downstream signaling pathways (30). Therefore, long-term EGFR-TKI therapy could potentially induce WMLs and GMA.

GMA and WMLs are reportedly associated with a rapid or excessive decline in global cognitive performance, executive function, and processing efficiency (17, 31, 32). Furthermore, structural changes in the brain are strongly correlated with a patient’s cognitive status (17, 33, 34). In the present study, the reduction in gray matter was nearly 0.92% after 12 months of EGFR-TKI treatment, while a large cross-sectional study (479 healthy participants) using SPM8 to measure age-related changes in GMV reported a global loss of 0.57% per year (35). Even though directly comparing these two studies is not sufficient evidence, it may be used to some extent to demonstrate the difference between patients receiving EGFR-TKI therapy and healthy people, especially given the difficulty in collecting longitudinal image data of healthy people along aging. Physical frailty-related GMV loss has been observed in the bilateral frontal and occipital cortices, while cognitive impairment-related GMV loss has been observed in the bilateral frontal, occipital, and temporal cortices (17). Similarly, we observed significant GMV loss mainly in sub-regions of the bilateral occipital lobes and the left basal ganglia. Unfortunately, no cognitive function tests (e.g., Wechsler adult intelligence scale-III, mini-mental state examination, color trails test, etc.) were performed on the patients who received EGFR-TKI treatment at baseline or during follow-up. We could not, therefore, analyze possible cognitive impairments caused by the changes in brain structure in the patients.

Previous studies on healthy elderly populations have reported either no significant progression of WMLs associated with age (36), or a mild increase of 0.2 to 0.4% per year (37), In this study, however, we observed significant deterioration of WMLs in patients with NSCLC who received EGFR-TKI therapy. The baseline WML score was significantly higher in older patients than in younger patients (p = 0.019), which is in accordance with recent reported findings (14, 22). At the 24-month follow-up, however, the WML scores were significantly higher in younger patients than older ones (p = 0.047), indicating that the younger patients were more sensitive to therapy. This could be explained by the higher proliferation rate of neural stem and progenitor cells in younger compared to older people, leading to a higher chance they would be affected by EGFR-TKI therapy. Women reportedly have significantly more WMLs relative to white matter volume than men (2.8 vs. 2.4%, respectively; p < 0.001) (38), and a greater marked progression of subcortical WML and incident lacunar infarcts than men (39). Similarly, the changes in WML scores in this study were more significant in female patients than in male patients at the 12-month and 24-month follow-ups. However, there were no significant differences in WML scores based on the type of EGFR mutation or EGFR-TKI therapy received.

For dementia research, visual rating scores from routine brain MRIs, which are recognized as a practical and inexpensive way to improve diagnostic accuracy, are recommended for assessing cognitive impairment. Prior to the application of more advanced image analysis techniques in clinical practice, visual rating scales were widely used and recommended for evaluating patients clinically with suspected dementia and was considered a diagnostic criterion for numerous types of dementia (22, 23, 40). Compared to several other visual rating scales, including the Fazekas scale (41), Rotterdam Scan Study (RSS) scale (37), modified SVS (24), Koedam posterior atrophy (PA) scale (42), and Prins scale (25), which were developed specifically to rate the vulnerability of brain regions to atrophy in different types of dementias, the SVS has been recommended for observing longitudinal changes in WMLs for chronic diseases and their relationship to clinical variables (43–45). GMV changes have been evaluated using the VBM-toolbox on SPM8, and the reliability of extracting quantitative brain metrics, such as GMV, in clinical-quality MRIs has been justified (46). Uncorrected voxel-based statistics increase the sensitivity as FDR increases (28). In this study, the FDR-corrected analysis was performed to minimize bias. Thus, the theoretical foundation and MRI analysis performed in the present study were relatively robust and have been validated by a large number of studies worldwide.

However, this study also had some limitations. First, the retrospective nature and relatively limited sample size of this study restricts its value in routine practice. Additionally, since a cognitive analysis was not conducted, interpreting the potential relationship between changes in the brain structure and cognition could not be assessed, even though significant worsening of the brain structure was observed. Second, data on mental status (depression or anxiety) before and during follow-up were not available, and subtle mental symptoms are difficult for patients to detect themselves. However, concomitant neuropsychiatric symptoms, such as depression or anxiety, in patients with NSCLC who undergo target therapy may exist, as reported previously (10). Third, systemic chemotherapy might have affected patients’ cognitive function (47, 48). Previous evaluations of cognition and brain structure changes in patients with lung cancer have demonstrated cognitive impairments after chemotherapy (49). Consequently, patients with lung cancer who undergo chemotherapy could not be used as controls. Additionally, no healthy volunteers were analyzed as controls in this retrospective setting. Therefore, the brain alterations observed in the present study should be interpreted cautiously unless they are validated by prospective data sets.

## Conclusion

This retrospective structural analysis of a series of brain MRIs showed significant worsening of WMLs and GMA in patients with advanced-stage NSCLC undergoing chronic EGFR-TKI treatment, which may indicate that this could be an unknown side-effect of EGFR-TKI treatment. Further prospective studies are being designed to more definitively determine the effects of long-term EGFR-TKI treatment on cognitive ability.

## Data Availability Statement

The raw data supporting the conclusions of this article will be made available by the authors, without undue reservation.

## Ethics Statement

The studies involving human participants were reviewed and approved by the ethics committee of West China Hospital, Sichuan University. The patients/participants provided their written informed consent to participate in this study. For patients were lost to follow-up (e.g., death, emigration), the informed consent waiver was permitted by the ethics committee at West China Hospital, Sichuan University.

## Author Contributions

YG conceived and designed the study. BY, CL, and YG collected, analyzed, and interpreted the data and drafted the article. BoT and BiT contributed to the evaluation of WML. MY, LZ, YiZ, JZ, MH, FP, YoL, YX, YaZ, XZ, JX, YaL, YW, ZL, YouL, and SL interpreted the data and revised the paper critically. All authors contributed to the article and approved the submitted version.

## Funding

This project was supported by grants from Sichuan Provincial Science and Technology Funding to YG (2018SZ0184) and LZ (2019YFS0323), and National Natural Science Foundation to LZ (81872466).

## Conflict of Interest

The author declares that the research was conducted in the absence of any commercial or financial relationships that could be construed as a potential conflict of interest.

The reviewer ZY declared a past co-authorship with one of the authors, SL, to the handling editor.

## Abbreviations

EGFR-TKI, Epidermal growth factor receptor-tyrosine kinase inhibitor; NSCLC, Non-small cell lung cancer; WML, white matter lesion; GMV, gray matter volume; MRI, magnetic resonance imaging; GMA, gray matter atrophy; VBM, voxel-based morphometry; FDR, false discovery rate; FLAIR, fluid attenuated inversion recovery.

## References

[1] Siegel RL Miller KD Jemal A Cancer statistics. CA Cancer J Clin (2018) 68:7–30. 10.3322/caac.21442 29313949

[2] Ciardiello F Tortora G A Novel Approach in the Treatment of Cancer: Targeting the Epidermal Growth Factor Receptor. Clin Cancer Res (2001) 7:2958. 11595683

[3] Holleman MS van Tinteren H Groen HJ Al MJ Uyl-de Groot CA First-line tyrosine kinase inhibitors in EGFR mutation-positive non-small-cell lung cancer: a network meta-analysis. Onco Targets Ther (2019) 12:1413–21. 10.2147/OTT.S189438 PMC6388947 30863108

[4] Loong HH Kwan SS Mok TS Lau YM Therapeutic Strategies in EGFR Mutant Non-Small Cell Lung Cancer. Curr Treat Pptions Oncol (2018) 19:58. 10.1007/s11864-018-0570-9 30267319

[5] Tan DS Yom SS Tsao MS Pass HI Kelly K Peled N The International Association for the Study of Lung Cancer Consensus Statement on Optimizing Management of EGFR Mutation-Positive Non-Small Cell Lung Cancer: Status in 2016. J Thorac Oncol (2016) 11:946–63. 10.1016/j.jtho.2016.05.008 27229180

[6] Rosell R Carcereny E Gervais R Vergnenegre A Massuti B Felip E Erlotinib versus standard chemotherapy as first-line treatment for European patients with advanced EGFR mutation-positive non-small-cell lung cancer (EURTAC): a multicentre, open-label, randomised phase 3 trial. Lancet Oncol (2012) 13:239–46. 10.1016/S1470-2045(11)70393-X 22285168

[7] David S Ettinger DEW Aggarwal C NCCN Clinical Practice Guidelines in Oncology: Non-Small Cell Lung Cancer NCCN guidelines Version 4. National Comprehensive Cancer Network (2019).

[8] Wu YL Planchard D Lu S Sun H Yamamoto N Kim DW Pan-Asian adapted Clinical Practice Guidelines for the management of patients with metastatic non-small-cell lung cancer: a CSCO-ESMO initiative endorsed by JSMO, KSMO, MOS, SSO and TOS. Ann Oncol (2019) 30:171–210. 10.1093/annonc/mdy554 30596843

[9] Shah RR Shah DR Safety and Tolerability of Epidermal Growth Factor Receptor (EGFR) Tyrosine Kinase Inhibitors in Oncology. Drug Saf (2019) 42:181–98. 10.1007/s40264-018-0772-x 30649743

[10] Kang HL Chen VC Hung WL Hsiao HP Wang WH Preliminary comparison of neuropsychological performance in patients with non-small-cell lung cancer treated with chemotherapy or targeted therapy. Neuropsychiatr Dis Treat (2019) 15:753–61. 10.2147/NDT.S194642 PMC6446983 31015761

[11] Goldshmit Y Greenhalgh CJ Turnley AM Suppressor of cytokine signalling-2 and epidermal growth factor regulate neurite outgrowth of cortical neurons. Eur J Neurosci (2004) 20:2260–6. 10.1111/j.1460-9568.2004.03698.x 15525267

[12] Kim IS Koppula S Park SY Choi DK Analysis of Epidermal Growth Factor Receptor Related Gene Expression Changes in a Cellular and Animal Model of Parkinson’s Disease. Int J Mol Sci (2017) 18(12):430. 10.3390/ijms18020430 PMC5343964 28212331

[13] Furnari FB Cloughesy TF Cavenee WK Mischel PS Heterogeneity of epidermal growth factor receptor signalling networks in glioblastoma. Nat Rev Cancer (2015) 15:302–10. 10.1038/nrc3918 PMC4875778 25855404

[14] Bolandzadeh N Davis JC Tam R Handy TC Liu-Ambrose T The association between cognitive function and white matter lesion location in older adults: a systematic review. BMC Neurol (2012) 12:126. 10.1186/1471-2377-12-126 23110387 PMC3522005

[15] Kloppenborg RP Nederkoorn PJ Geerlings MI van den Berg E Presence and progression of white matter hyperintensities and cognition: a meta-analysis. Neurology (2014) 82:2127–38. 10.1212/WNL.0000000000000505 24814849

[16] Li M Meng Y Wang M Yang S Wu H Zhao B Cerebral gray matter volume reduction in subcortical vascular mild cognitive impairment patients and subcortical vascular dementia patients, and its relation with cognitive deficits. Brain Behav (2017) 7:e00745. 10.1002/brb3.745 28828207 PMC5561307

[17] Chen YS Chen HL Lu CH Chen MH Chou KH Tsai NW Reduced lateral occipital gray matter volume is associated with physical frailty and cognitive impairment in Parkinson’s disease. Eur Radiol (2019) 29:2659–68. 10.1007/s00330-018-5855-7 30523452

[18] Prange S Metereau E Thobois S Structural Imaging in Parkinson’s Disease: New Developments. Curr Neurol Neurosci Rep (2019) 19:50. 10.1007/s11910-019-0964-5 31214847

[19] Dore V Villemagne VL Bourgeat P Fripp J Acosta O Chetelat G Cross-sectional and longitudinal analysis of the relationship between Abeta deposition, cortical thickness, and memory in cognitively unimpaired individuals and in Alzheimer disease. JAMA Neurol (2013) 70:903–11. 10.1001/jamaneurol.2013.1062 23712469

[20] Goldstein FC Mao H Wang L Ni C Lah JJ Levey AI White Matter Integrity and Episodic Memory Performance in Mild Cognitive Impairment: A Diffusion Tensor Imaging Study. Brain Imaging Behav (2009) 3:132–41. 10.1007/s11682-008-9055-y PMC2894481 20596297

[21] Therasse P Arbuck SG Eisenhauer EA Wanders J Kaplan RS Rubinstein L New guidelines to evaluate the response to treatment in solid tumors. European Organization for Research and Treatment of Cancer, National Cancer Institute of the United States, National Cancer Institute of Canada. J Natl Cancer Inst (2000) 92:205–16. 10.1093/jnci/92.3.205 10655437

[22] Kim KW MacFall JR Payne ME Classification of white matter lesions on magnetic resonance imaging in elderly persons. Biol Psychiatry (2008) 64:273–80. 10.1016/j.biopsych.2008.03.024 PMC2593803 18471801

[23] Valdes Hernandez MDC Chappell FM Munoz Maniega S Dickie DA Royle NA Morris Z Metric to quantify white matter damage on brain magnetic resonance images. Neuroradiology (2017) 59:951–62. 10.1007/s00234-017-1892-1 PMC5596039 28815362

[24] Scheltens P Barkhof F Leys D Pruvo JP Nauta JJP Vermersch P A semiquantative rating scale for the assessment of signal hyperintensities on magnetic resonance imaging. J Neurol Sci (1993) 114:7–12. 10.1016/0022-510X(93)90041-V 8433101

[25] Prins ND van Straaten EC van Dijk EJ Simoni M van Schijndel RA Vrooman HA Measuring progression of cerebral white matter lesions on MRI: visual rating and volumetrics. Neurology (2004) 62:1533–9. 10.1212/01.WNL.0000123264.40498.B6 15136677

[26] Davies RR Scahill VL Graham A Williams GB Graham KS Hodges JR Development of an MRI rating scale for multiple brain regions: comparison with volumetrics and with voxel-based morphometry. Neuroradiology (2009) 51:491–503. 10.1007/s00234-009-0521-z 19308367

[27] Ashburner J Friston KJ Voxel-based morphometry–the methods. NeuroImage (2000) 11:805–21. 10.1006/nimg.2000.0582 10860804

[28] Radua J Canales-Rodriguez EJ Pomarol-Clotet E Salvador R Validity of modulation and optimal settings for advanced voxel-based morphometry. NeuroImage (2014) 86:81–90. 10.1016/j.neuroimage.2013.07.084 23933042

[29] Lu H Cheng G Hong F Zhang L Hu Y Feng L A Novel 2-Phenylamino-Quinazoline-Based Compound Expands the Neural Stem Cell Pool and Promotes the Hippocampal Neurogenesis and the Cognitive Ability of Adult Mice. Stem Cells (Dayton Ohio) (2018) 36:1273–85. 10.1002/stem.2843 29726088

[30] Lichtenberger BM Tan PK Niederleithner H Ferrara N Petzelbauer P Sibilia M Autocrine VEGF signaling synergizes with EGFR in tumor cells to promote epithelial cancer development. Cell (2010) 140:268–79. 10.1016/j.cell.2009.12.046 20141840

[31] Prins ND van Dijk EJ den Heijer T Vermeer SE Jolles J Koudstaal PJ Cerebral small-vessel disease and decline in information processing speed, executive function and memory. Brain J Neurol (2005) 128:2034–41. 10.1093/brain/awh553 15947059

[32] Fujishima M Maikusa N Nakamura K Nakatsuka M Matsuda H Meguro K Mild cognitive impairment, poor episodic memory, and late-life depression are associated with cerebral cortical thinning and increased white matter hyperintensities. Front Aging Neurosci (2014) 6:306. 10.3389/fnagi.2014.00306 25426066 PMC4224123

[33] Garcia-Garcia R Cruz-Gomez AJ Mangas-Losada A Urios A Forn C Escudero-Garcia D Reduced resting state connectivity and gray matter volume correlate with cognitive impairment in minimal hepatic encephalopathy. PloS One (2017) 12:e0186463. 10.1371/journal.pone.0186463 29023586 PMC5638549

[34] Brickman AM Zahodne LB Guzman VA Narkhede A Meier IB Griffith EY Reconsidering harbingers of dementia: progression of parietal lobe white matter hyperintensities predicts Alzheimer’s disease incidence. Neurobiol Aging (2015) 36:27–32. 10.1016/j.neurobiolaging.2014.07.019 25155654 PMC4268124

[35] Yu T Korgaonkar MS Grieve SM Gray Matter Atrophy in the Cerebellum-Evidence of Increased Vulnerability of the Crus and Vermis with Advancing Age. Cerebellum (London England) (2017) 16:388–97. 10.1007/s12311-016-0813-x 27395405

[36] Matsusue E Sugihara S Fujii S Ohama E Kinoshita T Ogawa T White matter changes in elderly people: MR-pathologic correlations. Magn Reson Med Sci (2006) 5:99–104. 10.2463/mrms.5.99 17008766

[37] de Leeuw FE de Groot JC Achten E Oudkerk M Ramos LM Heijboer R Prevalence of cerebral white matter lesions in elderly people: a population based magnetic resonance imaging study. The Rotterdam Scan Study. J Neurol Neurosurg Psychiatry (2001) 70:9–14. 10.1136/jnnp.70.1.9 11118240 PMC1763449

[38] Fatemi F Kantarci K Graff-Radford J Preboske GM Weigand SD Przybelski SA Sex differences in cerebrovascular pathologies on FLAIR in cognitively unimpaired elderly. Neurology (2018) 90:e466–73. 10.1212/WNL.0000000000004913 PMC5818016 29343465

[39] van Dijk EJ Prins ND Vrooman HA Hofman A Koudstaal PJ Breteler MM Progression of cerebral small vessel disease in relation to risk factors and cognitive consequences: Rotterdam Scan study. Stroke (2008) 39:2712–9. 10.1161/STROKEAHA.107.513176 18635849

[40] Kipps CM Davies RR Mitchell J Kril JJ Halliday GM Hodges JR Clinical significance of lobar atrophy in frontotemporal dementia: application of an MRI visual rating scale. Dement Geriatr Cogn Disord (2007) 23:334–42. 10.1159/000100973 17374952

[41] Fazekas F Chawluk JB Alavi A Hurtig HI Zimmerman RA MR signal abnormalities at 1.5 T in Alzheimer’s dementia and normal aging. AJR Am J Roentgenol (1987) 149:351–6. 10.2214/ajr.149.2.351 3496763

[42] Koedam EL Lehmann M van der Flier WM Scheltens P Pijnenburg YA Fox N Visual assessment of posterior atrophy development of a MRI rating scale. Eur Radiol (2011) 21:2618–25. 10.1007/s00330-011-2205-4 PMC3217148 21805370

[43] Gouw AA van der Flier WM van Straaten EC Pantoni L Bastos-Leite AJ Inzitari D Reliability and sensitivity of visual scales versus volumetry for evaluating white matter hyperintensity progression. Cerebrovasc Dis (Basel Switzerland) (2008) 25:247–53. 10.1159/000113863 18216467

[44] Harper L Fumagalli GG Barkhof F Scheltens P O’Brien JT Bouwman F MRI visual rating scales in the diagnosis of dementia: evaluation in 184 post-mortem confirmed cases. Brain J Neurol (2016) 139:1211–25. 10.1093/brain/aww005 PMC4806219 26936938

[45] Robinson-Papp J Navis A Dhamoon MS Clark US Gutierrez-Contreras J Morgello S The Use of Visual Rating Scales to Quantify Brain MRI Lesions in Patients with HIV Infection. J Neuroimaging (2018) 28:217–24. 10.1111/jon.12466 PMC5821603 28833868

[46] Adduru VR Michael AM Helguera M Baum SA Moore GJ Leveraging Clinical Imaging Archives for Radiomics: Reliability of Automated Methods for Brain Volume Measurement. Radiology (2017) 284:862–9. 10.1148/radiol.2017161928 28448234

[47] Matsos A Johnston IN Chemotherapy-induced cognitive impairments: A systematic review of the animal literature. Neurosci Biobehav Rev (2019) 102:382–99. 10.1016/j.neubiorev.2019.05.001 31063740

[48] Hermelink K Buhner M Sckopke P Neufeld F Kaste J Voigt V Chemotherapy and Post-traumatic Stress in the Causation of Cognitive Dysfunction in Breast Cancer Patients. J Natl Cancer Inst (2017) 109(10):djx057. 10.1093/jnci/djx057 28521364

[49] Simó M Root JC Vaquero L Ripollés P Jové J Ahles T Cognitive and brain structural changes in a lung cancer population. J Thorac Oncol (2015) 10:38–45. 10.1097/JTO.0000000000000345 25325778 PMC5657249

