# Supplementary figures and images for: Changes of Brain Structure in Patients With Metastatic Non-Small Cell Lung Cancer After Long-Term Target Therapy With EGFR-TKI
Source: Front Oncol. 2021 Jan 6;10:573512. doi: 10.3389/fonc.2020.573512 (PMC7815525; doi:10.3389/fonc.2020.573512)

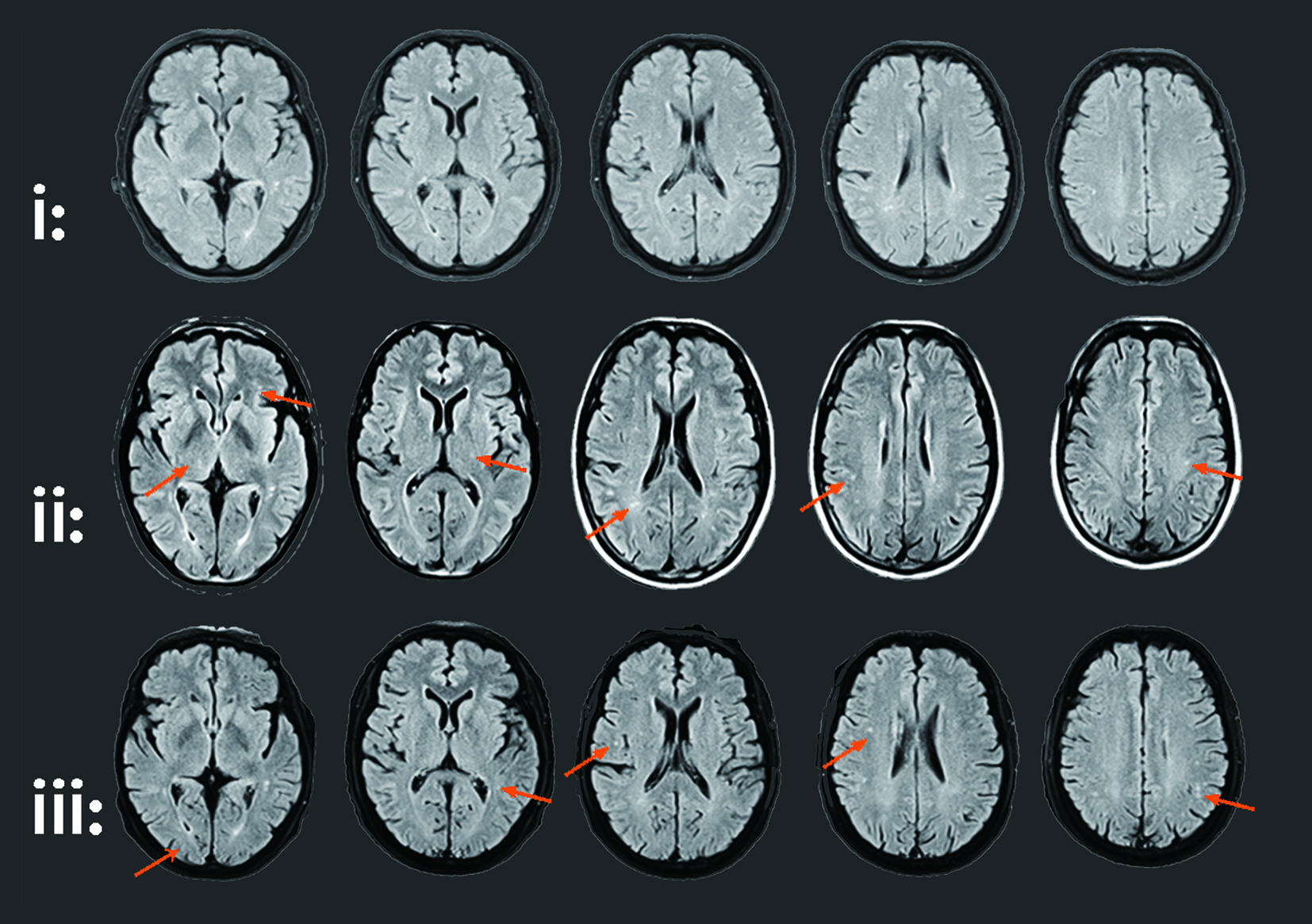

Supplement: Supplementary file 1 [file Image_1.tif]
