# Supplementary material for: Changes of Brain Structure in Patients With Metastatic Non-Small Cell Lung Cancer After Long-Term Target Therapy With EGFR-TKI
Source: Front Oncol. 2021 Jan 6;10:573512. doi: 10.3389/fonc.2020.573512 (PMC7815525; doi:10.3389/fonc.2020.573512)
Supplement: Supplementary file 2 [file Table_1.docx]

| The modified Scheltens visual scale*. | | |
| --- | --- | --- |
| Periventricular hyperintensitues (PVH 0-6) |  |  |
| Caps. occupital | 0/1/2 | 0 = absent |
| Frontal | 0/1/2 | 1= ≤5 mm |
| Bands lat ventricles | 0/1/2 | 2= >5 mm and <10 mm |
| Whtte matter hyperntensties (WMH 0-24) |  |  |
| Frontal | 0/1/2/3/4/5/6 | 0=na |
| Paretal | 0/1/2/3/4/5/6 | 1= ≤3mm, n≤5 |
| Occipital | 0/1/2/3/4/5/6 | 2= ≤3mm, n≥6 |
| Temporal | 0/1/2/3/4/5/6 | 3=4-10 mm, n≤5 |
|  |  | 4=4-10 mm, n≥6 |
|  |  | 5= ≥11mm, n≥1 |
|  |  | 6 = confluent |
| *Cited from Scheltens P, Barkhof F, Leys D, et al. A semiquantative rating scale for the assessment of signal hyperintensities on magnetic resonance imaging. Journal of the Neurological Sciences 1993;114:7-12. | | |
